# Metastatic Melanoma Invading the Minor Duodenal Papilla in a Patient With Pancreas Divisum Causing Acute Recurrent Pancreatitis: A Case Report

**DOI:** 10.7759/cureus.47543

**Published:** 2023-10-23

**Authors:** Barrett O Attarha, Gerardo Diaz Garcia, Andrew T Flint, Peter Senada, Bruno Ribeiro

**Affiliations:** 1 Gastroenterology, University of Florida College of Medicine - Jacksonville, Jacksonville, USA; 2 Internal Medicine, University of Florida College of Medicine - Jacksonville, Jacksonville, USA

**Keywords:** recurrent pancreatitis, ercp, endoscopic ultrasound (eus), endoscopic approach, ampulla, pancreas divisum, pancreatitis, melanoma

## Abstract

Metastasis to the gastrointestinal (GI) tract should always be a consideration when melanoma, particularly metastatic disease, is diagnosed. While metastasis to the small intestine is common, given its rich blood supply, metastasis to the pancreatic ducts is extremely rare. In patients with pancreatic divisum, disease spread to the minor papilla can greatly increase the chance of developing pancreatitis due to the potential for increased pancreatic intraductal pressure. We present one unique case of metastatic melanoma to the minor duodenal papilla causing pancreatitis.

## Introduction

Melanoma arises from the malignant transformation of specialized pigment-producing cells called melanocytes. Derived from neural crest cells, these cells are primarily found in the skin; however, they can also manifest in other parts of the body where neural crest cells migrate, such as the gastrointestinal (GI) tract and brain [1]. This type of cancer is highly aggressive, ranking as the third most common cutaneous malignancy and the most lethal among its counterparts. Prompt detection and treatment are crucial, as it has the potential for high mortality rates [2]. Fortunately, early identification offers a positive prognosis, with stage 0 cases showing an impressive 97% five-year survival rate. However, the survival rate for patients with metastasis is less than 20% after five years [1].

Metastasis most frequently affects the skin and subcutaneous tissue, followed by the lungs, liver, bones, and brain. Among carcinomas, malignant melanoma is the most common to metastasize to the GI tract. The small bowel is particularly susceptible (75% of all GI metastasis), followed by the colon (25% of all GI metastasis), liver (20% of all GI metastasis), and stomach (16% of all GI metastasis) [3,4]. Metastatic spread to the biliary system is rare, with only a limited number of reported cases [5,6]. Notably, no documented cases of melanoma metastasizing to the minor duodenal papilla have been reported. This report presents a unique case of metastatic melanoma affecting the minor duodenal papilla.

This case report was previously submitted for presentation as a meeting abstract at the 2020 ACG Annual Scientific Meeting on October 23, 2020 [7].

## Case presentation

**Figure 1:**
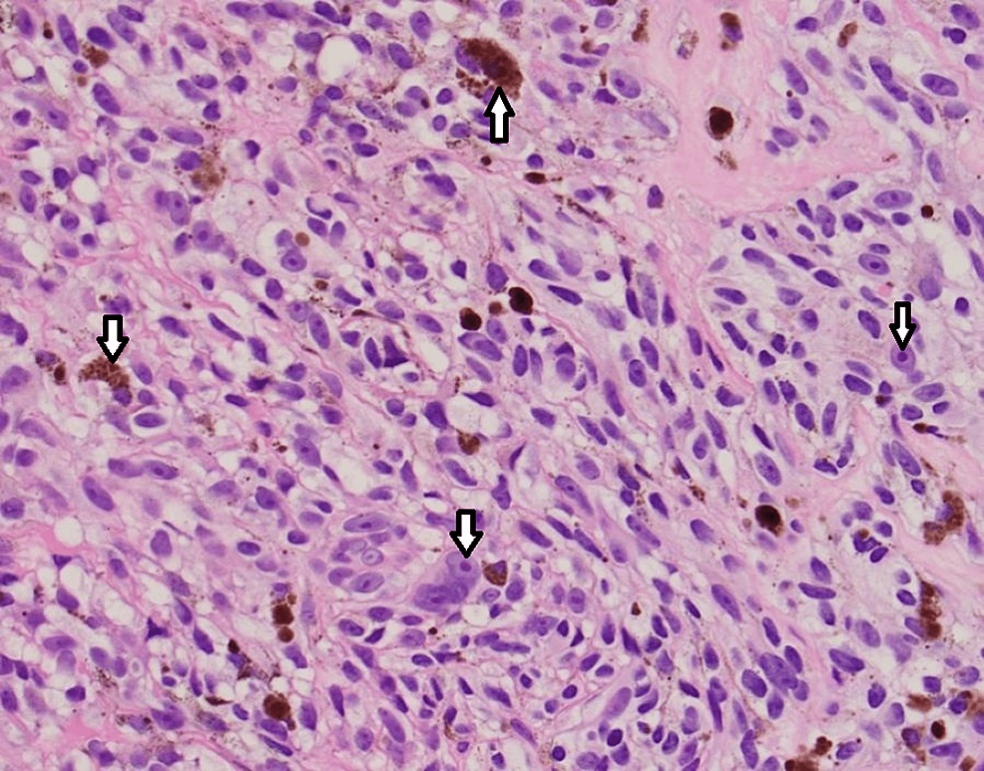
Tumor cells with scattered brown pigmentation. The tumor cells have large (so-called cherry red) nucleoli.

**Figure 2:**
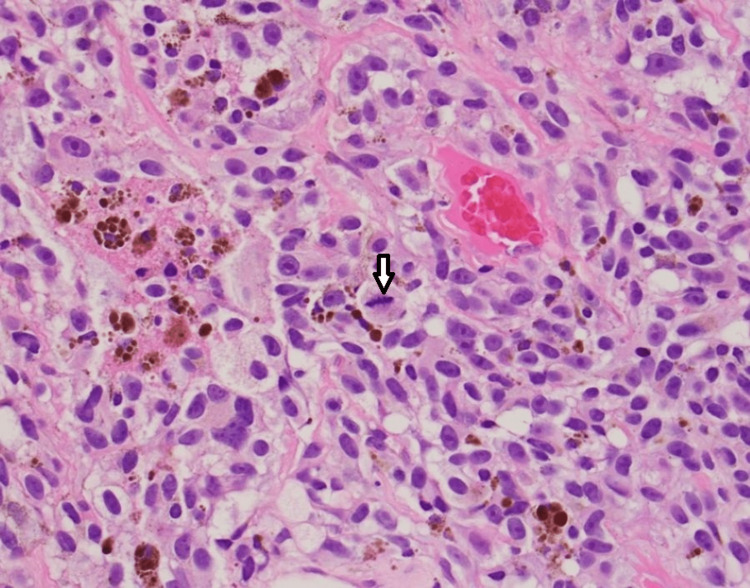
Tumor cells with a prominent mitotic figure in the center.

A 54-year-old male presented a third time to the Emergency Department with severe abdominal pain, lipase of 1654 U/L, and mildly elevated liver function tests. A computed tomography (CT) scan showed a metastatic process involving the entire pancreas, liver, and lungs, and also worsening pancreatic duct dilation (7 mm) compared to the previous CT scan. His past medical history is significant for melanoma of the right thumb status post amputation in 2008, tobacco abuse, and approximately 20 pounds of unintentional weight loss in three months. Three months earlier, when the metastatic disease was first detected, oncology was consulted and recommended a biopsy of a right axillary palpable lymph node which returned as metastatic melanoma v-raf murine sarcoma viral oncogene homolog B1 (BRAF) V600E negative (Figures 1, 2). He also had a magnetic resonance imaging (MRI) of the brain which showed multifocal intracranial metastases and completed whole brain radiation therapy with 30 Grays (Gy) in 10 fractions.

**Figure 3:**
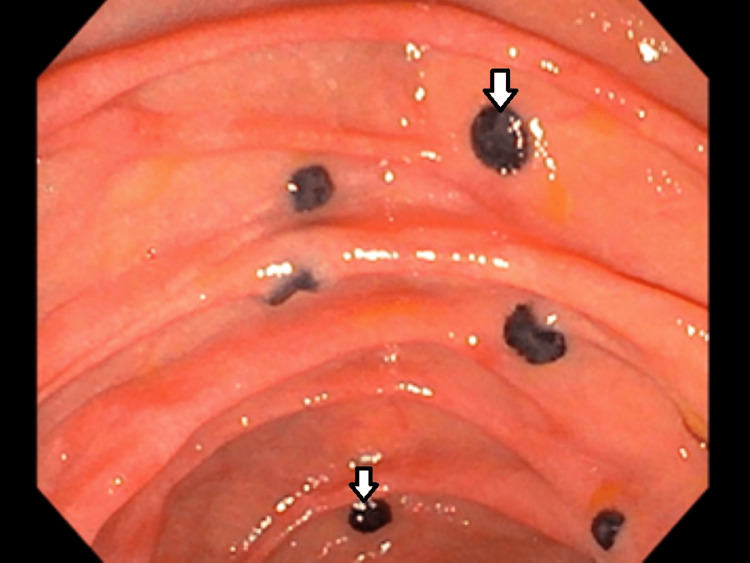
Flat black 3-4mm mass seen in duodenum representing metastatic melanoma.

**Figure 4:**
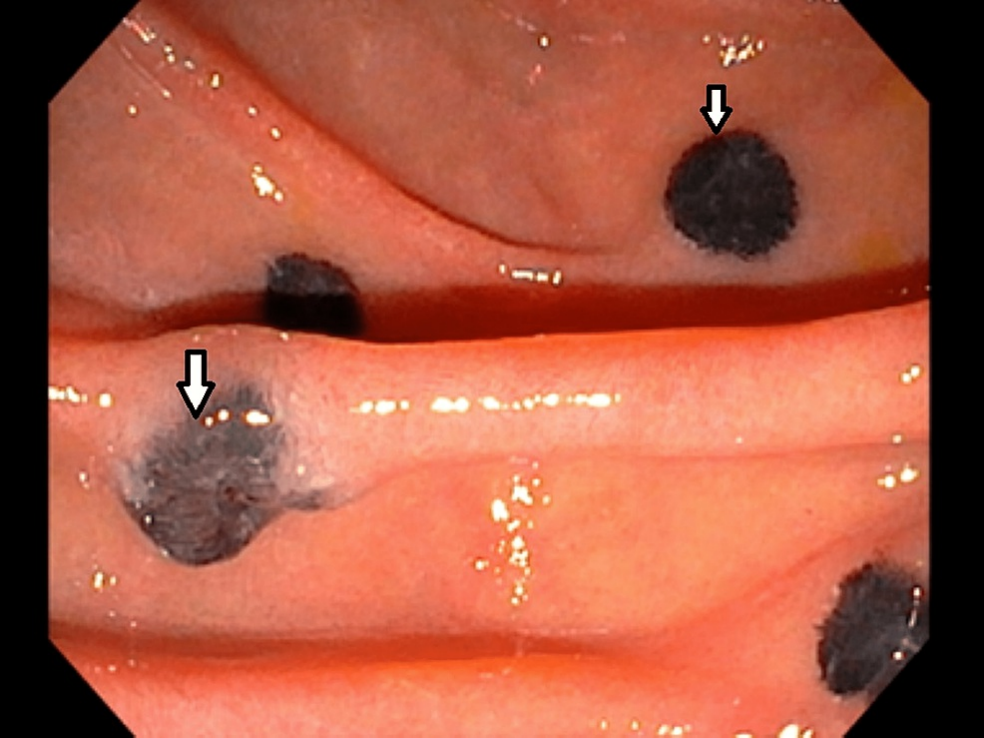
Close-up view of duodenal metastasis

**Figure 5:**
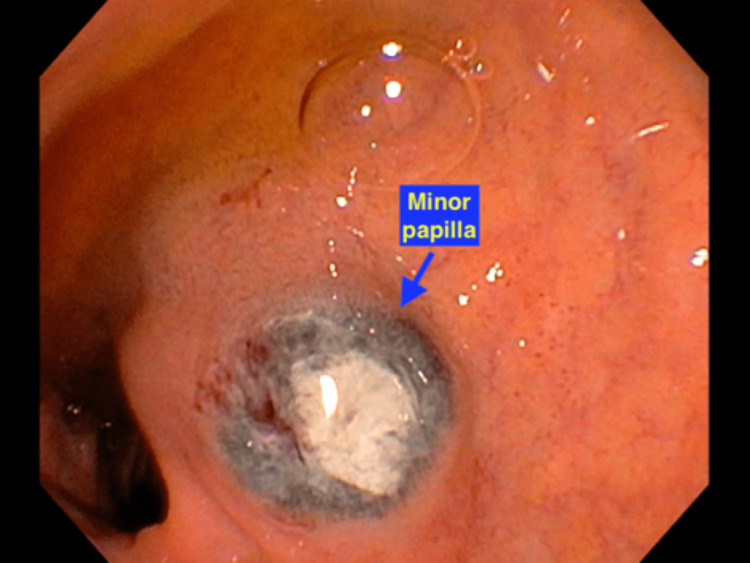
Melanotic lesion at the minor papilla

During the current admission, gastroenterology was consulted for acute recurrent pancreatitis, with one previous episode. They recommended endoscopic ultrasonography (EUS) and endoscopic retrograde cholangiopancreatography (ERCP). EUS detected multiple hypoechoic round and oval masses within the pancreatic and liver parenchyma suggesting metastasis. The EUS also revealed the pancreas divisum (PD), with the pancreatic duct measuring 5 mm in diameter. On the ERCP, the major papilla and the main biliary duct were normal. However, on the endoscopic view, many 3 to 10 mm black and flat infiltrative masses were detected in the first and second portions of the duodenum, suggestive of duodenal metastasis (Figures 3, 4). One of the flat masses was found in the minor papilla (Figure 5). The dorsal pancreatic duct could not be cannulated with the 3-4-5 tapered cannula and glidewire assistance. The patient improved with supportive care and was discharged in stable condition with a follow-up arranged with oncology for palliative treatment.

## Discussion

Metastatic melanoma metastasizes to the GI tract between 1% and 4% of patients with diagnosed metastatic malignant melanoma [8]. These are commonly found ante mortem and most often time occurs in the small intestine likely because of its highly vascular nature. This patient had multiple metastases visualized in the duodenum, a common site of tumor occurrence. Uncommon sites of metastasis include the esophagus, gallbladder, and biliary tree, with some cases having tumor cells with melanin found in bile [8]. This patient had multiple visualized small bowel tumors on ERCP; however, he did not have any signs or symptoms of GI bleeding, of note as this is a frequent presenting symptom of tumor spread [9]. Metastatic disease can, in some cases, be severe enough to cause small bowel perforation as reported in one case by Tsilimparis et al. [10]. There have also been reported cases of melanoma spreading to the pancreas, including one instance of primary anorectal melanoma [11]. No cases of melanoma metastasizing to the minor papilla causing acute recurrent pancreatitis, as in this case report, were found in the literature. The patient also has a PD, previously undiagnosed and seen on EUS, which likely predisposed him to the development of acute pancreatitis [12].

PD is the most common pancreatic developmental anatomic variant with a prevalence of up to 11% in Western populations and is usually asymptomatic [12]. These patients have a failure of the dorsal and ventral pancreatic buds fusing and thus result in a majority of the pancreas draining through the dorsal duct of Santorini through the minor papilla. The abnormal fusion causes abnormal drainage of the majority of the pancreatic juice into the naturally narrowed minor papilla, causing the elevation of the intraductal pressure in some patients [13,14]. Among other causes of acute pancreatitis, stenosis of the minor papilla can be coexistent in PD [15]. Acute recurrent pancreatitis can be related to PD in up to 30% of cases [16]. Experimental studies in canine models demonstrated that the functional obstruction of the minor papilla at the peak stage of secretion and total ligation of the ventral duct, like PD, can be an etiological factor for pancreatitis [17].

## Conclusions

This patient had no prior episodes of pancreatitis, exocrine or endocrine insufficiency, and no abdominal pain prior to his metastatic melanoma diagnosis, making the melanotic lesion the likely culprit in this case for acute pancreatitis. The patient presented with a dilated pancreatic duct; however, many patients with PD have a dilated pancreatic duct and remain asymptomatic. Therefore, it is hypothesized that the duodenum melanotic lesion caused some obstruction at the minor papilla and may have aggravated the already increased ductal pressure with PD leading to recurrent pancreatitis in this patient.

## References

[1] Heistein JB, Acharya U, Mukkamalla SKR (2023). Malignant Melanoma. http://www.ncbi.nlm.nih.gov/books/NBK470409/.

[2] Sundararajan S, Thida AM, Yadlapati S, Koya S (2023). Metastatic Melanoma. https://www.ncbi.nlm.nih.gov/books/NBK470358/.

[3] Kohoutova D, Worku D, Aziz H, Teare J, Weir J, Larkin J (2021). Malignant melanoma of the gastrointestinal tract: symptoms, diagnosis, and current treatment options. Cells.

[4] Leung AM, Hari DM, Morton DL (2012). Surgery for distant melanoma metastasis. Cancer J.

[5] Piltcher-da-Silva R, Sasaki VL, Hutten DO (2021). Biliary tract melanoma metastasis mimicking hilar cholangiocarcinoma: a case report. J Surg Case Rep.

[6] van Bokhoven MM, Aarntzen EH, Tan AC (2006). Metastatic melanoma of the common bile duct and ampulla of Vater. Gastrointest Endosc.

[7] Attarha B, Stemboroski L, De Souz Ribeiro B (2020). A unique case of acute pancreatitis due to metastatic melanoma to the minor papilla in a patient with pancreas divisum. Am J Gastroenterol.

[8] Blecker D, Abraham S, Furth EE, Kochman ML (1999). Melanoma in the gastrointestinal tract. Am J Gastroenterol.

[9] Colombo E, Bocchi M, Giorgi S (2002). GI metastases from melanoma: a case report. Am J Gastroenterol.

[10] Tsilimparis N, Menenakos C, Rogalla P, Braumann C, Hartmann J (2009). Malignant melanoma metastasis as a cause of small-bowel perforation. Onkologie.

[11] Dai JJ, Qu CS, Wang W, Wang YB, Mao XW, Li QS, Chen JF (2020). Primary anorectal malignant melanoma: a case report. Int J Clin Exp Pathol.

[12] Covantev S (2018). Pancreas divisum: a reemerging risk factor for pancreatic diseases. Rom J Intern Med.

[13] Kozu T, Suda K, Toki F (1995). Pancreatic development and anatomical variation. Gastrointest Endosc Clin North Am.

[14] Staritz M, Meyer zum Büschenfelde KH (1988). Elevated pressure in the dorsal part of pancreas divisum: the cause of chronic pancreatitis?. Pancreas.

[15] Warshaw AL, Simeone JF, Schapiro RH, Flavin-Warshaw B (1990). Evaluation and treatment of the dominant dorsal duct syndrome (pancreas divisum redefined). Am J Surg.

[16] Chalazonitis NA, Lachanis BS, Laspas F, Ptohis N, Tsimitselis G, Tzovara J (2008). Pancreas divisum: magnetic resonance cholangiopancreatography findings. Singapore Med J.

[17] He H, Lu WF, Ke YZ, Zhang YM (1998). An experimental study in etiologic effect of pancreas divisum on chronic pancreatitis and its pathogenesis. World J Gastroenterol.

